# HCCS1-armed, quadruple-regulated oncolytic adenovirus specific for liver cancer as a cancer targeting gene-viro-therapy strategy

**DOI:** 10.1186/1476-4598-10-133

**Published:** 2011-11-01

**Authors:** Hai-Neng Xu, Wei-Dan Huang, Ying Cai, Miao Ding, Jin-Fa Gu, Na Wei, Lan-Ying Sun, Xin Cao, Hua-Guang Li, Kang-Jian Zhang, Xin-Ran Liu, Xin-Yuan Liu

**Affiliations:** 1 State Key Laboratory of Cell Biology, Institute of Biochemistry and Cell Biology, Shanghai Institutes for Biological Sciences, Chinese Academy of Sciences, Shanghai 200031, China; 2 Xinyuan Institute of Medicine and Biotechnology, Zhejiang Sci-Tech University, Hangzhou 310018, China

**Keywords:** liver cancer, quadruple regulated adenovirus, HCCS1, mitochondrial apoptosis pathway

## Abstract

**Background:**

In previously published studies, oncolytic adenovirus-mediated gene therapy has produced good results in targeting cancer cells. However, safety and efficacy, the two most important aspects in cancer therapy, remain serious challenges. The specific expression or deletion of replication related genes in an adenovirus has been frequently utilized to regulate the cancer cell specificity of a virus. Accordingly, in this study, we deleted 24 bp in E1A (bp924-bp947) and the entirety of E1B, including those genes encoding E1B 55kDa and E1B19kDa. We used the survivin promoter (SP) to control E1A in order to construct a new adenovirus vector named Ad.SP.E1A(Δ24).ΔE1B (briefly Ad.SPDD). HCCS1 (hepatocellular carcinoma suppressor 1) is a novel tumor suppressor gene that is able to specifically induce apoptosis in cancer cells. The expression cassette AFP-HCCS1-WPRE-SV40 was inserted into Ad.SPDD to form Ad.SPDD-HCCS1, enabling us to improve the safety and efficacy of oncolytic-mediated gene therapy for liver cancer.

**Results:**

Ad.SPDD showed a decreased viral yield and less toxicity in normal cells but enhanced toxicity in liver cancer cells, compared with the cancer-specific adenovirus ZD55 (E1B55K deletion). Ad.SPDD-HCCS1 exhibited a potent anti-liver-cancer ability and decreased toxicity in vitro. Ad.SPDD-HCCS1 also showed a measurable capacity to inhibit Huh-7 xenograft tumor growth on nude mice. The underlying mechanism of Ad.SPDD-HCCS1-induced liver cancer cell death was found to be via the mitochondrial apoptosis pathway.

**Conclusions:**

These results demonstrate that Ad.SPDD-HCCS1 was able to elicit reduced toxicity and enhanced efficacy both in vitro and in vivo compared to a previously constructed oncolytic adenovirus. Ad.SPDD-HCCS1 could be a promising candidate for liver cancer therapy.

## Background

Liver cancer is one of the most common malignant tumors worldwide and the third leading cause of cancer-related death. Because of its high malignancy and fast progression, most patients with high-grade cancers have tumors that are unresectable. To date, no remedy has shown efficacy in these patients [1,2]. It is important that we develop an efficient and safe drug for patients with liver cancer.

The strategy of "Cancer Targeting Gene-Viro-Therapy" (CTGVT), was developed in 2001 [3] and has shown promise in the treatment of cancer. It combines the advantages of gene therapy and oncolytic viral therapy. Oncolytic adenoviruses can replicate themselves in cancer cells and lyse the carcinoma, but very inefficient in normal cells. Tumor suppressor genes can replicate together with oncolytic viral vectors, thereby greatly enhancing the ability to induce cancer cell death [4]. The advantage of this combination is to enable the oncolytic adenovirus to harbor antitumor genes, thereby facilitating them to specifically and efficiently kill cancer cells. Cancer Targeting Dual Gene-Viro-Therapy (CTGVT-DG) with a combination of two viruses carrying compensatory or synergetic genes has shown near eradication of tumors in nude mice [5-7]. Cancer Targeting Gene-Viro-Therapy Specific for Liver Cancer (CTGVT-LC) is a modification of CTGVT that can specifically target liver cancer. It is predicted that excellent antitumor drugs will arise from CTGVT and its modifications but are unlikely to result from gene or viral therapy alone [8].

Adenoviruses have been modified in various ways to improve their specificity in cancer cells. In such viruses, E1A, an early adenovirus gene, is controlled by cancer specific promoters, such as the survivin promoter [9], to regulate specific replication of adenoviruses. The CR2 domain of E1A (bp924- bp947) is known to interact with the Rb protein, repressing Rb activation and increasing the replication of the adenovirus. Deletion of this 24 bp domain results in the adenovirus selectively targeting various cancer cells with dysfunction of the Rb pathway [10,11]. The E1B 55kDa gene has been deleted to form the cancer-specific adenovirus ONYX-015 [12]. It is now believed that E1B 55kDa functions as a result of late viral RNA export rather than p53 inactivation, as was previously believed [13]. Heat shock can cause phenocopy of E1B 55KDa late function in most cancer cells [14]. However, when an adenovirus is modified, its anti-tumor efficacy is usually reduced. The E1B 19kDa protein was deleted in previous studies to enhance the anti-tumor efficacy of an adenovirus, specifically its ability to inhibit cell apoptosis by blocking the function of the proapoptotic proteins Bax, Bak, p53 and TNF [15].

α-fetoprotein (AFP) is an established marker of liver cancer, and it is present at increased levels in the serum of approximately 75% of all liver cancer patients [16]. The high levels of AFP are an indication of the specific activity of the AFP promoter in liver cancer, and it may be used to improve the safety of gene therapy in AFP positive liver cancer cells. AFP promoter was used to control the anti-tumor gene cytosine deaminase and cytosine deaminase specially expressed in liver cancer cells [17]. The adenovirus with the key replicative gene E1A under the control of AFP promoter can specially replicate in AFP positive liver cancer cell lines [18,19]. All this indicates that AFP promoter as a potential regulatory element in liver cancer therapy.

HCCS1 was discovered as a liver cancer specific tumor suppressor gene that is frequently mutated in liver cancer [20,21]. A previously constructed oncolytic adenovirus, ZD55-HCCS1, has been shown to elicit strong anti-tumor efficacy [22]. WPRE (Woodchuck Hepatitis Virus Posttranscriptional Regulatory Element) is a cis-acting RNA element that is capable of increasing the expression of genes located upstream [23] and can be used in various kinds of vectors to promote gene expression [24].

In this study, we constructed a new oncolytic adenovirus vector by deleting 24 bp of E1A, as well as the genes encoding E1B 55kDa and E1B 19kDa. We also utilized a survivin promoter(denoted as SP) to control E1A, and named it as Ad.SP.E1A(Δ24).ΔE1B (also denoted Ad.SPDD). This quadruple-regulated adenovirus, Ad.SPDD, exhibited improved safety and enhanced efficacy compared with ZD55 (similar to ONYX-015), an adenovirus with an E1B 55kD deletion that was previously developed in our laboratory showing capacity to target cancer cells [4-7,25,26]. Moreover, this adenovirus with complete E1B deletion permits the insertion of large gene expression cassettes.

To enhance both anti-tumor efficacy and specificity, the HCCS1 expression cassette AFP-HCCS1-WPRE-SV40 was introduced into the vector Ad.SP.E1A(Δ24).ΔE1B to produce Ad.(AFP-HCCS1-WPRE)SP.E1A(Δ24).ΔE1B (also denoted Ad.SPDD-HCCS1). Additionally, owing to the lack of a commercial primary antibody specific for HCCS1, another adenovirus, Ad.SPDD-HCCS1HA, in which HCCS1 is fused to an HA tag named HCCS1HA, was developed at the same time to detect the expression of the HCCS1 protein. Ad.SPDD-HCCS1 showed a potent anti-tumor effect specific for liver cancer in both in vitro and in vivo experiments, with induction of the mitochondrial apoptosis pathway as its underlying mechanism. Our CTGVT-LC strategy using Ad.SPDD-HCCS1 may be a promising therapy for selectively targeting liver cancer.

## Results

### Liver cancer-specific transcriptional activity of the AFP promoter

**Figure 1:**
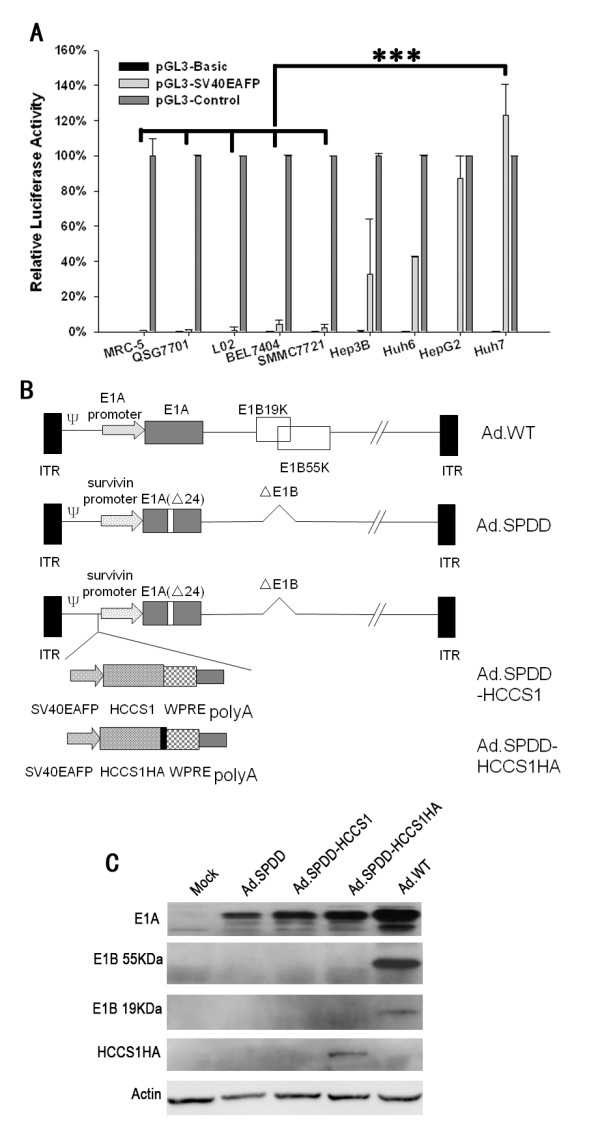
**Construction and characterization of viruses**. (A) Transcriptional activity of the AFP promoter in different cell lines. The AFP promoter with an SV40 enhancer was incorporated into pGL3-basic. The relative activity of pGL3-SV40EAFP to pGL3-Control in various cell lines are presented as the mean+SD (n = 3). (B) Schematic structure of the viruses Ad.SPDD, Ad.SPDD-HCCS1, and Ad.SPDD-HCCS1. ITR, inverted terminal repeat; ψ, viral packing signal. Ad.SPDD was quadruply modified compared to the wild-type adenovirus; the modifications were the deletion of E1B19K, the deletion of E1B55K, and the deletion of 24 bp of E1A, as well as deletion of the survivin promoter controlling E1A expression. HCCS1 or HA-tagged HCCS1 in a cassette was carried by Ad.SPDD. (C) Characterization of viruses by western blot analysis. Huh-7 cells were infected with the indicated viruses, and the levels of the expressed E1A, E1B 19KDa, E1B 55KDa and HA-Tag were analyzed 48 hours post infection (h.p.i), with actin as loading control. (***: P < 0.001).

The transcriptional activity of the commercialized SV40EAFP in different cell lines was detected using a luciferase reporter assay. In addition to being present in three normal cell lines, SV40EAFP activity was observed in four AFP positive and two AFP negative liver cancer cell lines. As shown in Figure 1A, the SV40EAFP promoter exhibited 33%-123% as high activity as the pGL3-control in the AFP positive cell lines Hep3B, Huh-6, HepG2 and Huh-7, whereas this activity was only 1%-4% as high in the AFP negative cell lines BEL7404 and SMMC7721, as well as in the normal cell lines MRC-5, QSG7701 and L-02. These results suggested that SV40EAFP was especially active in AFP positive liver cancer cell lines.

### Virus construction and characterization

The quadruple-regulated adenoviruses Ad.SPDD, Ad.SPDD-HCCS1, and Ad.SPDD-HCCS1HA were constructed as shown in Figure 1B. Virus identification utilizing PCR amplification and sequencing of the PCR products of E1A confirmed the insertion of the gene expression cassettes and the deletion of 24 bp of E1A, as well as that the virus stocks were free of wild-type adenovirus contamination (Additional file 1). Western blotting was also performed to detect adenovirus gene expression in Huh-7 cells (Figure 1C). All viruses expressed E1A, whereas all viruses except for the wild-type adenovirus (Ad.WT) failed to express E1B 19KDa and E1B 55KDa. Anti-HA antibody detection revealed the expression of HCCS1, which was observed only for Ad.SPDD-HCCS1HA. The HCCS1HA gene was expressed at particularly high levels in Huh-7 cells, as observed via the detection of HA tag-specific antibody expressed in Ad.SPDD-HCCS1HA-infected MRC-5 and Huh-7 cells (Additional file 2).

### Ad.SPDD exhibited less toxicity and an enhanced ability to lyse cancer cells than ZD55

**Figure 2:**
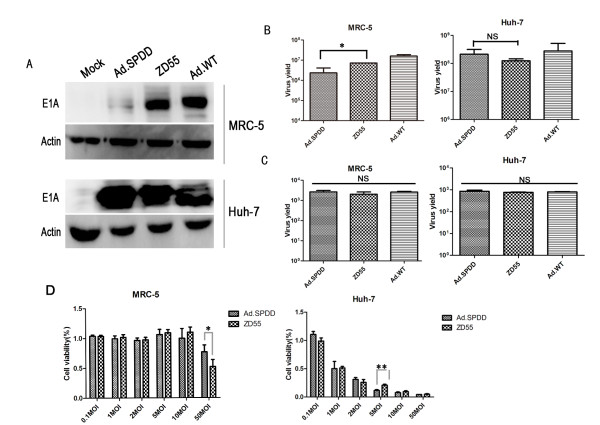
**Ad.SPDD shows less toxicity and an enhanced efficacy at lysing cancer cells compared to ZD55**. (A) Specific expression of E1A in Huh-7 cells infected with Ad.SPDD. MRC-5 and Huh-7 cells were infected with Ad.SPDD, ZD55 and Ad.WT or without any infection, and the expression of E1A was detected 48 hours post infection, with actin used as loading control. (B) The replicative ability of the adenoviruses in MRC-5 and Huh-7 cells. MRC-5 and Huh-7 cells were infected at an MOI of 10, and the adenovirus titer was measured 48 hours after infection. The results are presented as the mean+SD (n = 3). (C) The adenovirus titer 8 hours after adenovirus infection of MRC-5 and Huh-7 cells. The adenovirus titers in the MRC-5 and Huh-7 cells were measured 8 hours after infection with the indicated adenovirus at an MOI of 10. (D) Ad.SPDD showed improved cancer specificity over ZD55. MRC-5 and Huh-7 cells were infected with Ad.SPDD and ZD55 at the indicated MOIs. Cell viability was measured 4 days post infection. The results are presented as the mean+SD (n = 6). * indicates P < 0.05, *** indicates P < 0.001, and NS means not significant (with a P value > 0.05).

The levels of E1A expressed by Ad.SPDD, ZD55, and Ad.WT in MRC-5 and Huh-7 cells were detected by western blot analysis. The expression of E1A by ZD55 and Ad.WT in MRC-5 cells was strong, although only traces of E1A were observed to be expressed by Ad.SPDD. However, there was no significant difference in E1A expression by Ad.SPDD, ZD55 and Ad.WT in Huh-7 cells, indicating that the survivin promoter can specifically control the expression of E1A by Ad.SPDD in cancer cells (Figure 2A).

The replicative ability of Ad.SPDD, ZD55, and Ad.WT in both normal and cancer cell lines was tested. Ad.SPDD replicated nearly as efficiently as ZD55 and Ad.WT in Huh-7 and Hep3B cells, but its replication was weaker in MRC-5 cells (Figure 2B, Additional file 3). In MRC-5 and Huh-7 cells, the adenovirus titer at 8 hours after infection was used as negative control, and it showed very low titer compared to the titer at 48 hours post infection (Figure 2C). A cell viability assay was performed to examine the cytotoxicity of Ad.SPDD and ZD55 on MRC-5 and Huh-7 cells. Ad.SPDD showed lower toxicity than did ZD55 on MRC-5 cells at an MOI of 50, whereas it exhibited higher cytotoxicity than ZD55 on Huh-7 cells at an MOI of 5 (Figure 2D). These results demonstrated the reduced toxicity and enhanced efficacy associated with the quadruple-regulated adenovirus Ad.SPDD compared with ZD55.

### Ad.SPDD-HCCS1 specifically targeted liver cancer in vitro

**Figure 3:**
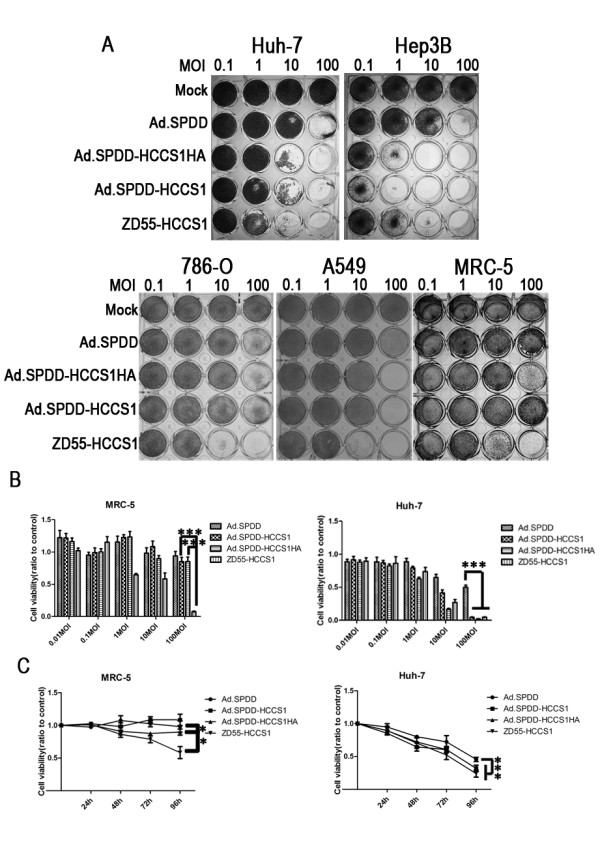
**Ad.SPDD-HCCS1 has specific anti-liver-cancer activity in vitro**. (A) Crystal violet staining was performed on both tumor and normal cells that had been infected with adenovirus at the indicated MOIs. AFP positive cancer cells (Huh-7 and Hep3B), AFP negative cancer cells (786-O and A549) and AFP negative normal cells (MRC-5) were infected with various adenoviruses at an MOI of 0.1, 1, 10, and 100. Crystal violet staining was performed 4 days after infection. (B) Viability of the MRC-5 and Huh-7 cells infected at various viral MOIs on the 4th day. (C) Viability of MRC-5 and Huh-7 cells infected at an MOI of 10 at the indicated time. The cells were infected with different viruses and were measured from days 1 to 4. Results are presented as the mean ± SD for 6 repeated wells. A P value < 0.05 or < 0.01 is indicated as * or **, respectively.

The cytotoxicity of the viruses against both normal and cancer cell lines was evaluated by a crystal violet staining assay and an MTT assay. As shown in Figure 3A, the cytopathic effects of Ad.SPDD-HCCS1 and Ad.SPDD-HCCS1HA were equal to that of ZD55-HCCS1 in the AFP positive liver cancer cell lines Huh-7 and HepG2. The cytopathic effects were lower in the AFP-negative cancer cell lines 786-O, A549, and the normal cell line MRC-5, as expected. This finding suggests that Ad.SPDD-HCCS1 possesses a higher specificity for AFP positive liver cancer than does ZD55-HCCS1. Cell viability was measured either 4 days after viral infection at different MOIs (Figure 3B) or was detected each day till the 4th day after a viral infection at an MOI of 10 (Figure 3C). The Ad.SPDD-HCCS1 and Ad.SPDD-HCCS1HA showed potent cytotoxicity against Huh-7 cells, similar to ZD55-HCCS1, and elicited time-dependent and dose-dependent effect. However, both the adenoviruses showed little cytotoxicity on MRC-5 cells. Notably, both in the crystal violet staining assay and the MTT assay, ZD55-HCCS1 damaged the normal cell line MRC-5 to some extent after infection at an MOI of 10, indicating a potential safety hazard with clinical use at high doses. However, Ad.SPDD-HCCS1 had no observable cytotoxic effect even at an MOI of 100.

### Antitumor efficacy of Ad.SPDD-HCCS1 on tumor xenografts

**Figure 4:**
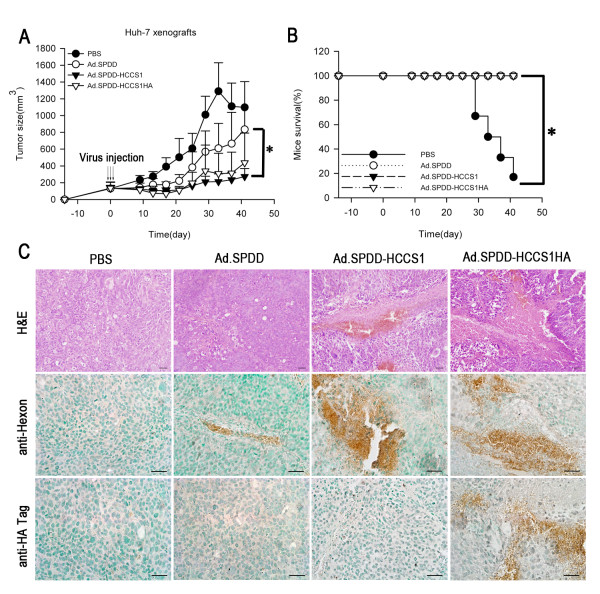
**Antitumor efficacy of viruses on Huh-7 tumor xenografts in nude mice**. (A) Ad.SPDD-HCCS1 and Ad.SPDD-HCCS1HA suppress the growth of Huh-7 tumors in vivo. The tumor volumes (mean+SD, n = 6) were measured and estimated every four days. (B) The ratios of surviving mice during the observation period in groups treated with different adenoviruses. (C) H&E staining and immunohistochemical staining of tumor sections using anti-hexon and anti-HA tag antibodies. Representative images are shown at 400 × magnification for the different samples Scale bar = 100 μm. (*: P < 0.05).

In addition to its liver cancer-specific cytotoxicity in vitro, the antitumor efficacy of the new construct was also evaluated in vivo on Huh-7 tumor xenografts that had been established in nude mice. These tumors had a high degree of malignancy and grew rapidly. In the PBS group, most mice either had tumor sizes of more than 2000 mm^3 ^or died within 2 months. At the end of the experiment, only one nude mouse remained in the PBS group, and this mouse was very weak. However, the nude mice in the Ad.SPDD, Ad.SPDD-HCCS1 and Ad.SPDD-HCCS1HA groups were all alive. The mean tumor volume of the Ad.SPDD, Ad.SPDD-HCCS1 and Ad.SPDD-HCCS1HA groups was 835.93 mm^3^, 272.60 mm^3 ^and 436.99 mm^3^, respectively (Figures 4A and 4B), indicating that our newly designed Ad.SPDD-HCCS1 and Ad.SPDD-HCCS1HA constructs were able to strongly inhibit liver cancer growth.

To further determine whether the gene or virus can efficiently exist in the tumor tissue and induce tumor suppression, tumor sections from the Ad.SPDD-, Ad.SPDD-HCCS1- and Ad.SPDD-HCCS1HA-treated mice were stained with hematoxylin and eosin (H&E) and analyzed. As shown in Figure 4C, there were large necrotic regions in the tumor sections from Ad.SPDD-HCCS1- and Ad.SPDD-HCCS1HA-treated mice. Smaller necrotic areas were also observed in the tumor sections from Ad.SPDD-treated mice. We examined the expression of the adenovirus capsid protein hexon and HCCS1 (via an HA-tag-specific antibody) by immunohistochemical staining, and we found significant positive staining by hexon-specific antibody in the adenoviruses treated groups, but only Ad.SPDD-HCCS1HA infected tumor shows positive staining by HA tag-specific antibody (Figure 4C). Compared with the negative result in the PBS group, both the viral replication and HCCS1 expression in the tumor xenograft suggested that the efficacy of tumor suppression may have resulted from the function of both the virus and the expressed gene.

### Ad.SPDD-HCCS1 induced apoptosis of liver cancer cells both in vitro and in vivo

**Figure 5:**
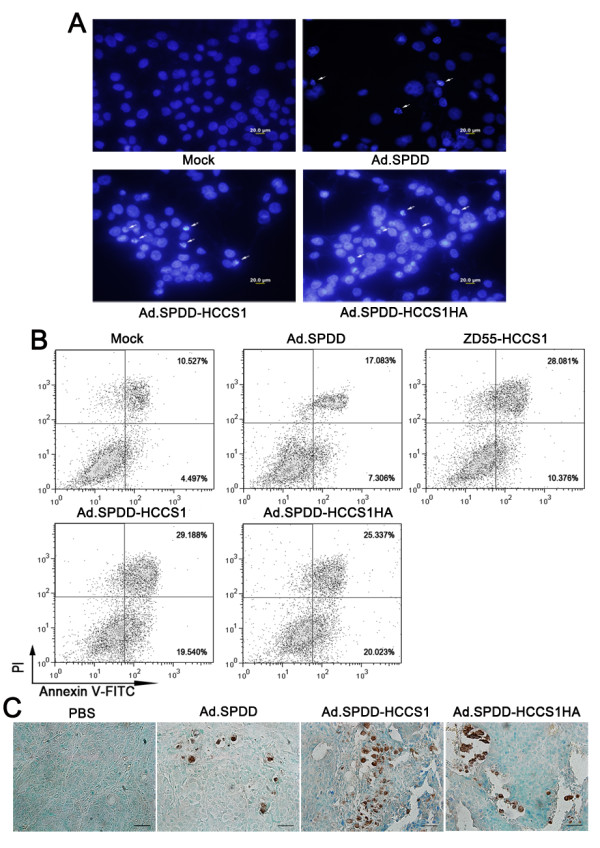
**Ad.SPDD-HCCS1 can induce apoptosis of hepatocellular carcinoma cells both in vitro and in vivo**. (A) Ad.SPDD-HCCS1 and Ad.SPDD-HCCS1HA induce more nuclear condensation than Ad.SPDD. Hoechst33258 staining was performed on Huh-7 cells 48 hours after infection with viruses at an MOI of 10. Scale bar = 20 μm. (B) Annexin V-FITC/PI staining FACS assay. The percentages of the cells in the second and fourth quadrants are shown. (C) Detection of apoptosis in tumor sections by TUNEL staining. Scale bar = 100 μm.

Because Ad.SPDD-HCCS1 effectively inhibited tumor growth, several assays were performed to investigate the underlying mechanism. As shown in Figure 5A, infection with Ad.SPDD-HCCS1 apparently caused nuclear fragmentation and chromatin condensation in the Huh-7 cells. When the virus-infected tumor cells were subjected to flow cytometric assay after Annexin V-FITC/PI staining, we found that the percentage of Annexin-V positive cells in the Ad.SPDD-HCCS1- or Ad.SPDD-HCCS1HA-infected cell samples was similar to that in the ZD55-HCCS1-infected cell sample, and all of these cell samples had a higher percentage of Annexin-V positive cells than the mock- and Ad.SPDD- infected cell samples (Figure 5B). In addition to this in vitro test, virus-treated tumor xenografts were sectioned and analyzed for apoptosis. TUNEL analysis revealed that Ad.SPDD-HCCS1 and Ad.SPDD-HCCS1HA each elicited profound apoptosis in the tumors (Figure 5C), whereas no apoptosis was observed in sections from PBS-treated mice. Taken together, the results showed that the anti-tumor characteristics of the virus resulted partly from its induction of apoptosis.

### Ad.SPDD-HCCS1 induced apoptosis of liver cancer cells via the mitochondrial/caspase-9/caspase-3 pathway

**Figure 6:**
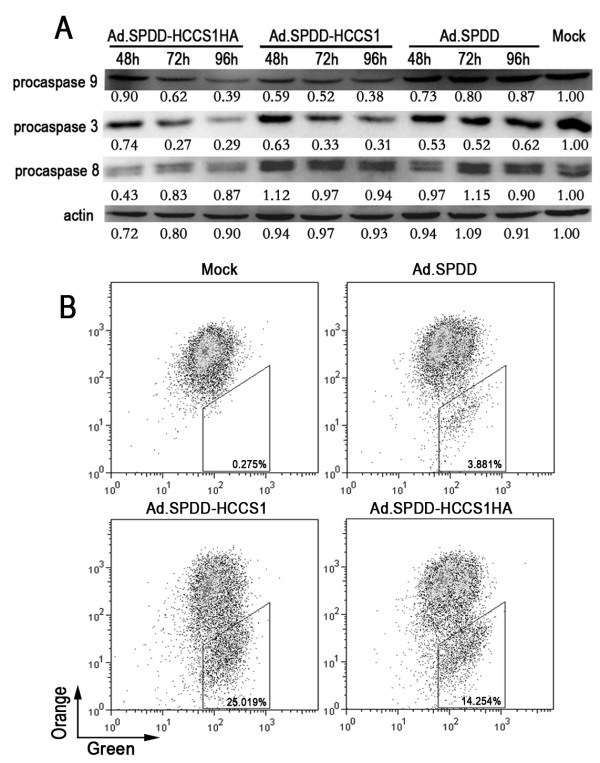
**Ad.SPDD-HCCS1 induces mitochondrial-mediated apoptosis in liver cancer cells**. (A) Western blot analysis of apoptosis-related proteins in Huh-7 cells infected with Ad.SPDD, Ad.SPDD-HCCS1 or Ad.SPDD-HCCS1HA. Actin was used as a loading control. (B) Ad.SPDD-HCCS1 and Ad.SPDD-HCCS1HA induced an increased loss of mitochondrial membrane potential in Huh-7 cells. The mitochondrial membrane potentials of the Huh-7 cells infected with the indicated viruses were analyzed by JC-1 staining via FACS detection. The percentage of cells in the trapeziform region is shown as cells with lost mitochondrial membrane potential.

To further understand the mechanism by which Ad.SPDD-HCCS1 caused apoptosis of liver cancer cells, we evaluated the key apoptosis-related proteins caspase-3, caspase-8 and caspase-9. As shown in Figure 6A, the precursors of caspase-3 and caspase-9 were present in decreased amounts, indicating the activation of caspase-3 and caspase-9, 48 hours after viral infection of Huh-7 cells compared with before viral infection. Little difference in the amount of the caspase-8 precursor that was present was shown even 96 hours post virus treatment compared with before viral infection, indicating that the apoptosis induced by Ad.SPDD-HCCS1 may occur via the mitochondrial pathway. We further tested the changes in the mitochondrial membrane potential, Δψ_m_, of the virus-infected cells with a JC-1 probe. A marked increase in JC-1 monomers was found in Ad.SPDD-HCCS1- and Ad.SPDD-HCCS1HA- treated Huh-7 cells, indicating a drop in Δψm, but not in Ad.SPDD-infected Huh-7 cells (Figure 6B). These results suggested that Ad.SPDD-HCCS1 induced the apoptosis of liver cancer cells via the mitochondrial pathway.

## Discussion

In this study, we successfully constructed a quadruple-regulated adenovirus named Ad.SPDD-HCCS1 with specificity for liver cancer cells. This adenovirus was generated with the following: the survivin promoter (SP) driving the E1A gene; the deletion of both of the Rb binding domains of E1A; the whole of E1B, including the genes encoding E1B-19K and E1B-55K; and the liver cancer-specific expression cassette AFP-HCCS1-WPRE-SV40, which was inserted upstream of E1A. Although a dual- or triple-regulated vector was studied in previous studies, to our knowledge no quadruple-regulated adenoviral vectors have previously been reported.

It is well known that modifying an adenovirus to improve its specificity usually reduces the replication capacity of the adenovirus while lowering its antitumor efficacy. It is important to achieve an effective balance between safety and efficacy when designing an adenovirus to target liver cancer. The quadruple-regulated adenovirus Ad.SPDD satisfied both specificity and efficacy. It showed improved replication in cancer cells and enhanced antitumor effects on Huh-7 cells compared to ZD55, along with a much lower toxicity than ZD55 against the normal cell line MRC-5 according to the MTT assay. The reduced toxicity might result in a better safety profile in vivo. Moreover, the complete deletion of E1B in Ad.SPDD allowed space for the insertion of an expression cassette with large genes.

The cytotoxicity and anti-tumor capacity of Ad.SPDD-HCCS1 were detected in vitro and in vivo, respectively; its anti-liver-cancer activity was as good as that of ZD55-HCCS1. More significantly, Ad.SPDD-HCCS1 exhibited decreased toxicity than ZD55-HCCS1, as shown by both a crystal violet staining assay and an MTT assay, suggesting that Ad.SPDD-HCCS1 is a significantly better construct than ZD55-HCCS1 for liver cancer targeting. In the Figure 3C it seems that the Ad.SPDD-HCCS1 and Ad.SPDD-HCCS1HA elicited similar effect as the Ad.SPDD, although indeed they showed significant difference, with the P value smaller than 0.001. However, in Figure 3B, Huh-7 cells infected with Ad.SPDD-HCCS1, Ad.SPDD-HCCS1HA at an MOI of 100 showed a lot of difference with Ad.SPDD. This is due to the dose-dependent effect of the adenoviruses, the HCCS1 gene carried by adenovirus exhibited its function in the condition of sufficient expression.

The AFP promoter was used in this work to drive HCCS1 to improve its safety. As many patients with liver carcinoma have liver cirrhosis, but not normal liver, and some liver cirrhosis are AFP positive. Yao F et al. found that the percentage of AFP positive samples in patients with liver cirrhosis is smaller than that with liver cancer, and the AFP mRNA in samples of liver cirrhosis is lower than that of liver cancer [27]. This indicated the special AFP promoter activity in liver cancer cells, though with slight activity in cirrhosis. Otsuru A et al. found that in liver cirrhosis sections only a small number of cells showed positive hybridization by in situ hybridization analysis of AFP mRNA, but the majority of tumor cells showed positive hybridization [28]. This indicates that AFP promoter may be a specific anti-liver-cancer cell promoter, and combines it with other safety regulatory element will possibly improve its safety further.

In the Ad.SPDD-HCCS1 or Ad.SPDD-HCCS1HA, there are two promoters, survivin promoter driving E1A and AFP promoter driving HCCS1 or HCCS1HA, in the adjacent region, it is possible that they may cis activate each other with the enhancers. However, in Ad.SPDD, the survivin promoter used is just 269 bp, a very short promoter, and it possesses essential sites for binding transcription factors [29]. It should not affect the AFP promoter. As to the enhancer on the AFP promoter's effect on survivin promoter, Figure 3B and 3C preclude this possibility. Ad.SPDD-HCCS1 or Ad.SPDD-HCCS1HA didn't show any enhanced cytoxicity on MRC-5 compared to Ad.SPDD. This indicated two issue, one is that HCCS1 is specially expressed in the Huh-7 cells, which is demonstrated by additional file 2 and another one is that the AFP-HCCS1-SV40 cassette didn't enhance the cytotoxicity of the vector adenovirus Ad.SPDD, indicating that the survivin promoter activity is not elevated in MRC-5 by AFP promoter. In the hypothesis that the survivin promoter is enhanced by the AFP promoter, Ad.SPDD-HCCS1 or Ad.SPDD-HCCS1HA will show more cytoxicity on MRC-5 than Ad.SPDD due to the elevated replicative ability in MRC-5, but this is opposite to the shown results.

Ad.SPDD-HCCS1 may specifically inhibit liver cancer in two ways. First, the adenovirus replicated in the liver cancer cells caused lysis. Second, the amplification of the tumor suppressor gene HCCS1 was accompanied by replication of the oncolytic adenoviral vector, while it also induced apoptosis through the mitochondrial/caspase-9/caspase-3 pathway.

The mechanism by which Ad.SPDD-HCCS1 kills liver cancer cells was demonstrated to be mitochondria-triggered apoptosis. Despite detecting apoptosis both in vitro and in vivo, the level of ATP in the infected cells increased. This is not consistent with the phenomenon of apoptosis, in which the cellular ATP level decreases. It is known that, of the three main types of programmed cell death, only autophagy leads to an increase in cellular ATP levels. It has been reported that autophagy occurs in the progression of adenoviral infection of cells. Therefore, we proposed that autophagy might have contributed to the inhibitory effect of Ad.SPDD-HCCS1on liver cancer cells. To test this hypothesis, we co-infected Huh-7 cells with an LC3-EGFP plasmid and Ad.SPDD-HCCS1, but no punctate fluorescence was observed (data not shown). In addition, the apoptosis rate measured by the Annexin V-FITC/PI flow cytometric assay and the death rate measured by the MTT cell viability test were similar (approximately 30 percent). Thus, we cannot confirm the involvement of autophagy in this phenomenon, although we speculate that necrosis-like cell death, as reported by Abou El Hassan MA [30], may be occurring. Some non-classical programmed cell death phenomena may also have occurred as a result of viral replication and transgene expression. Because Ad.SPDD-HCCS1 treatment increased the total ATP level without altering the rate of cell death, it is possible that viral replication plays a role.

Ad.SPDD-HCCS1 showed a potent and specific anti-liver cancer efficacy. As such, we believe that Ad.SPDD-HCCS1 could bee a promising candidate for the treatment of AFP positive liver cancer.

## Conclusions

Quadruple-regulated adenoviruses carrying an AFP promoter-controlled HCCS1 gene showed reduced toxicity and excellent anti-liver cancer efficacy in this study. The adenoviral vector Ad.SPDD had less toxicity and more efficacy than the previously constructed cancer-specific adenovirus ZD55. The gene-armed oncolytic adenovirus Ad.SPDD-HCCS1 exhibited liver-cancer specificity and observable anti-liver-cancer capacity both in vitro and in vivo. This work provides a feasibility study for generating effective cancer specificity and anti-cancer efficacy by using an adenoviral vector with a combination of modifications. Using the Ad.SPDD-HCCS1 construct seems to be a promising strategy for future clinical application in liver cancer therapy.

## Methods

### Cell lines and cell culture

The human liver cancer cell lines Huh-7 and Huh-6 were obtained from the RIKEN Cell Bank (Ibaraki, Japan). The human liver cancer cell lines HepG2, Hep3B and BEL7404; the human colorectal cancer cell line SW620; the human renal carcinoma cell line 786-O; the human normal liver cell lines L-02 and QSG-7701; and the human lung fibroblast cell line MRC-5 were purchased from the Cell Bank of the Type Culture Collection of the Chinese Academy of Sciences (Shanghai, China). The human embryonic kidney 293 cell line (HEK293) was obtained from Microbix Biosystems Inc. (Toronto, Ontario, Canada). All cell lines were cultured according to the instructions of the corresponding providers.

### Luciferase Reporter Assay

The pGL3-Basic and pGL3-Control reporter plasmids were purchased from Promega (Madison, WI), whereas the pCMV-β-gal normalization plasmid was obtained from Invitrogen (Carlsbad, CA). The AFP promoter with an SV40 enhancer, SV40EAFP, was purchased from Invivogen (San Diego, CA) and cloned into pGL3-Basic to form the reporter plasmid pGL3-SV40EAFP. Cells were seeded in 24-well plates at 80% confluence and transfected with reporter plasmids and pCMV-β-gal using Lipofectamine 2000 (Invitrogen). Cells were harvested 48 hours later, and the luciferase activity was detected using the Luciferase Assay System (Promega).

### Plasmid and virus construction

The adenoviral packaging vector pBHGE3 was obtained from Microbix Biosystem Inc. The adenoviral shuttle vectors pCA13Δ, pAd.surP-E1AΔ24, and pAd.AFP-E1A-ΔE1B had previously been constructed by our group pAd.surP-E1AΔ24 and pAd.AFP-E1A-ΔE1B were digested with EcoRI and XbaI, respectively, to obtain surP-E1AΔ24 and pAd. ΔE1B, respectively. These were ligated to form pAd.SP. ΔE1B. The promoters SV40EAFP and WPRE were inserted into pCA13Δ by using the restriction enzymes Sal I and BamHI, respectively. HCCS1 was amplified by PCR using forward (5'-CGGGAATTCATGATGGAGGAGGAGGAA-3') and reverse (5'-TTAGGATCCCTCGAGCTACGTCCATCTCACCTG-3') primers. HCCS1HA was amplified by PCR using forward (5'-CGGGAATTCATGATGGAGGAGGAGGAA-3') and reverse (5'-TTACTCGAGTTATCAGGCGTAGTCGGGACGTCGTAGGGGTAC- GTCC ATCTCACCTGTTC -3') primers, with ZD55-HCCS1 as the template. The PCR products were digested with EcoRI and XhoI and cloned into pCA13-AFP-WPRE to construct pCA13-HCCS1 and pCA13-HCCS1HA. The HCCS1 and HCCS1HA expression cassettes were released and introduced into pAd.SPDD to produce pAd.SPDD-HCCS1 and pAd.SPDD-HCCS1HA. The adenoviruses Ad.SPDD, Ad.SPDD-HCCS1 and Ad.SP.DD-HCCS1HA were generated by homologous recombination between the corresponding plasmid and pBHGE3 in the HEK293 cells, using the Effectene Transfection Reagent (Qiagen, Germany) as the transfection reagent. The viruses were amplified in HEK293 cells and purified by ultracentrifugation in a cesium chloride gradient. The titers of the viruses were measured in HEK293 cells.

### Adenovirus replication assay in vitro

MRC-5, Huh-7 and Hep3B cells were seeded in 6-well plates at a density of 1 × 10^5 ^per well and infected with Ad.SPDD, ZD55 or wild-type adenovirus at an MOI of 10. The cells were washed with PBS 3 times 8 hours post-infection and fresh medium was added. All cells and the culture medium were collected 48 hours post-infection, and the adenoviruses were titered, after being released by three freeze-thaw cycles. MRC-5 and Huh-7 cells with the indicated adenoviruses infected for 8 hours were also collected after washing with PBS for 3 times, the cells were titered after three freeze-thaw cycles as a negative control to 48 hours' infected cells.

### Western blot assay

Cells were treated with different agents at an MOI of 10 and harvested at the indicated times. Western blot analysis was carried out according to standard procedures. E1A-, caspase-3-, and actin-specific antibodies and secondary antibodies were obtained from Santa Cruz Biotechnology (Santa Cruz, CA). Caspase-8- and caspase-9-specific antibodies were purchased from Cell Signaling Technology (Beverly, MA). The E1B 55kDa-specific antibody was purchased from Oncogene (Cambridge, MA), the E1B 19kDa-specific antibody was purchased from Calbiochem (San Diego, CA), and the Hexona-specific antibody was obtained from Millipore (Billerica, MA).

### Animal experiments

The animal experiments were conducted according to the U.S. Public Health Service Policy on Humane Care and the Use of Laboratory Animals. The experiments were also approved by the Institutional Animal Care and Use Committee. Four-week-old male BALB/c nude mice were purchased from the Shanghai Experimental Animal Center (Shanghai, China). A total of 8 × 10^6 ^Huh-7 cells was injected subcutaneously in the right rear back of each mouse to establish the xenograft tumor. When the tumor size reached 100-150 mm^3^, the mice were randomly divided into 4 groups and intratumorally injected with adenovirus (5 × 10^8 ^PFU per mouse each time) or PBS four times in the first 5 days, with no virus injected on the third day. Subsequently, the lengths and widths of the tumors were measured every four days. Tumor volume (mm^3^) was calculated as length × (width)^2^/2.

### Immunohistochemical analysis and TdT-mediated dUTP nick end labeling assays

Tumor tissues were collected four days post-injection, fixed in 4% formaldehyde overnight, and embedded in paraffin. Subsequently, 5- μm-thick sections were cut and subjected to H&E staining, immunohistochemistry and TdT-mediated dUTP nick end labeling assay (TUNEL). The expression of hexon and HCCS1 with an HA Tag were detected separately using a goat anti-hexon (Chemicon) and a rabbit anti-HA (Santa Cruz) antibody, respectively. The immunohistochemistry assay was performed using the rabbit or goat SP (HRP) staining system (Westang Biotechnology, Shanghai, China). The TUNEL assay was performed according to the manufacturer's instructions (R&D systems).

### Cytotoxicity assay

For the crystal violet staining, the cells were infected with the various viruses at the indicated MOI. Four days later, the cells were stained with 2% crystal violet in 20% methanol for 1 h, then washed carefully and photographed.

For the 3-(4, 5-dimethylthiazol-2-yl)-2, 5-diphenyl tetrazolium bromide (MTT) assay, cells infected with viruses at different MOI were cultured at 37°C for 4 d after infection, then 20 μl of MTT solution (Sigma) was added per well (5 mg/ml) and the cells were incubated at 37°C for 4 h. The supernatant was then removed, and 100 μl of 0.04 M HCl-isopropanol solution was added to each well. Cell viability was measured by absorbance at dual wavelengths (595 nm and 650 nm) using a Microplate Reader (Bio-Rad). The viability of cells infected with viruses at an MOI of 10 was detected in the same way on days 1 to 4.

### Hoechst33258 staining

Cells were infected with different viruses at an MOI of 10. Forty-eight hours later, the cells were fixed with 4% paraformaldehyde, stained with Hoechst33258 (Molecular Probes, Eugene, OR) at 1 μg/ml for 1 min, and then visualized with a microscope.

### Flow cytometric analysis

Cells were infected with various viruses at an MOI of 10, and both adherent and suspended cells were collected 48 hours post-infection. Detection of apoptosis was performed following the manufacturer's instructions for the Annexin V-FITC Apoptosis Detection kit (BioVision). For analysis of the mitochondrial membrane potential, the collected cells were stained with the JC-1 fluorescent probe (Beyotime, Nantong, China) and analyzed using a FACSCalibur flow cytometer.

### Statistical analysis

All data were analyzed using Student's t test or a one-way analysis of variance (ANOVA) using the software R and subsequently shown as the mean+SD. The P value results are shown as P < 0.05:*, P < 0.01:**, or P < 0.001:*** in the figures. A one-way ANOVA was performed using R software when there were more than two groups in the comparison analysis. The TukeyHSD command was also used to comparatively assess the P values between two independent groups. The survival curves were created in Sigma plot, and the P values were calculated using GraphPad Prism 5.0 using the log-rank test.

## List of abbreviations

Ad.SPDD: Ad.SP.E1A(Δ24).ΔE1B; HCCS1: Hepatocellular carcinoma suppressor 1; CTGVT: Cancer Targeting Gene-Viro-Therapy; CTGVT-LC: Cancer Targeting Gene-Viro-Therapy Specific for Liver Cancer; AFP: α-fetoprotein; WPRE: Woodchuck Hepatitis Virus Posttranscriptional Regulatory Element; TUNEL: TdT-mediated dUTP nick end labeling assay; and MTT: 3-(4, 5-dimethylthiazol-2-yl)-2; 5-diphenyl tetrazolium bromide.

## Competing interests

The authors declare that they have no competing interests.

## Authors' contributions

XHN, HWD and LXY designed and wrote the study. HWD and ZKJ constructed the plasmids. HWD, GJF, and SLY produced and identified the viruses. WN and LHG amplified and purified the viruses. XHN, HWD, CY, DM, and LXR performed the cytotoxic experiments. XHN, HWD, and CX did the animal experiments. XHN, HWD, and LXY performed the statistical analysis and data interpretation. All authors read and approved the manuscript.

## Supplementary Material

Additional file 1 **Identification of the viruses at the DNA level**. (A) Blast sequence results for the region covering the deleted 24 bp of E1A (B) Identification of the E1B region of the viruses in order to detect wild type adenovirus contamination, by PCR amplification assay. Ad.WT and ZD55 virus DNA were tested as positive controls. (C)-(E) PCR results of positions at which the HCCS1 gene cassette, HCCS1 gene and SV40EAFP promoter were inserted. pAd.SPDD-HCCS1HA was used as positive control. pAd.SPDD was tested as negative control. Click here for file

Additional file 2 **HCCS1HA was specifically expressed in Huh-7 cells**. MRC-5 and Huh-7 cells were infected with Ad.SPDD-HCCS1HA at an MOI of 10. The cells were collected for western blot 48 hours post infection. An anti-HA antibody was used to detect the expression of HCCS1HA. Actin was used as a loading control. Click here for file

Additional file 3 **Ad.SPDD, ZD55, and Ad.WT exhibit similar replicative ability in Hep3B cells**. Hep3B cells were infected with Ad.SPDD, ZD55, or Ad.WT at an MOI of 10. The adenoviral titers were measured 48 hours post infection. (NS: not significant, p > 0.05) Click here for file
