# Supplementary figures and images for: HCCS1-armed, quadruple-regulated oncolytic adenovirus specific for liver cancer as a cancer targeting gene-viro-therapy strategy
Source: Mol Cancer. 2011 Nov 1;10:133. doi: 10.1186/1476-4598-10-133 (PMC3222618; doi:10.1186/1476-4598-10-133)

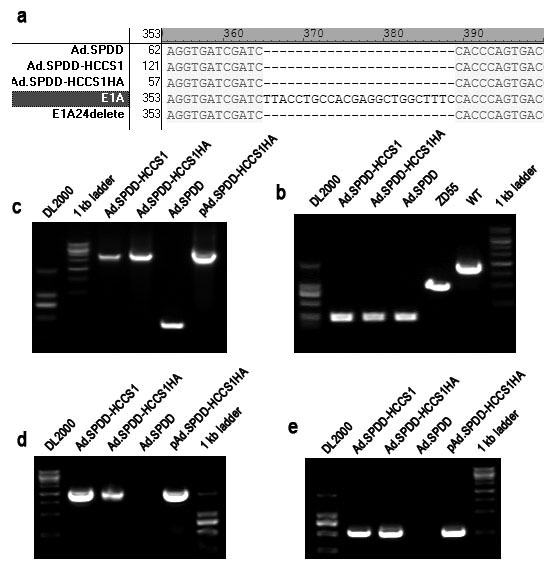

Supplement: Additional file 1 — Identification of the viruses at the DNA level. (A) Blast sequence results for the region covering the deleted 24 bp of E1A (B) Identification of the E1B region of the viruses in order to detect wild type adenovirus contamination, by PCR amplification assay. Ad.WT and ZD55 virus DNA were tested as positive controls. (C)-(E) PCR results of positions at which the HCCS1 gene cassette, HCCS1 gene and SV40EAFP promoter were inserted. pAd.SPDD-HCCS1HA was used as positive control. pAd.SPDD was tested as negative control. [file 1476-4598-10-133-S1.JPEG]

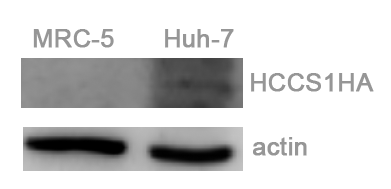

Supplement: Additional file 2 — HCCS1HA was specifically expressed in Huh-7 cells. MRC-5 and Huh-7 cells were infected with Ad.SPDD-HCCS1HA at an MOI of 10. The cells were collected for western blot 48 hours post infection. An anti-HA antibody was used to detect the expression of HCCS1HA. Actin was used as a loading control. [file 1476-4598-10-133-S2.TIFF]

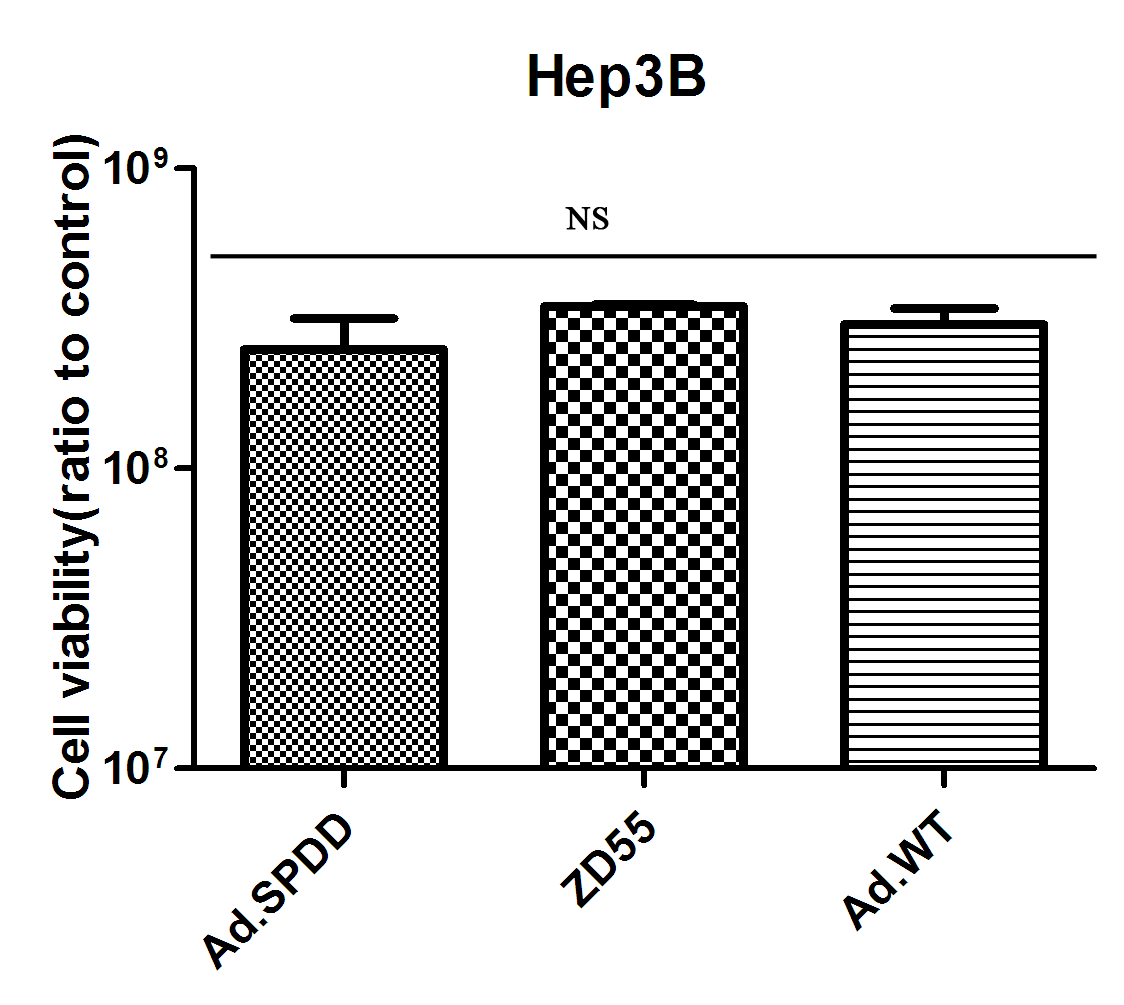

Supplement: Additional file 3 — Ad.SPDD, ZD55, and Ad.WT exhibit similar replicative ability in Hep3B cells. Hep3B cells were infected with Ad.SPDD, ZD55, or Ad.WT at an MOI of 10. The adenoviral titers were measured 48 hours post infection. (NS: not significant, p > 0.05) [file 1476-4598-10-133-S3.TIFF]
